# How to diagnose cervicogenic dizziness

**DOI:** 10.1186/s40945-017-0040-x

**Published:** 2017-09-12

**Authors:** Alexander S. Reiley, Frank M. Vickory, Sarah E. Funderburg, Rachel A. Cesario, Richard A. Clendaniel

**Affiliations:** Doctor of Physical Therapy Division, Duke University, 2200 W Main St., Durham, NC 27705 USA

**Keywords:** Cervicogenic dizziness, Cervical dizziness, Disequilibrium, Vertigo, Cervical spine, Cervical proprioception, Whiplash, Diagnosis

## Abstract

Cervicogenic dizziness (CGD) is a clinical syndrome characterized by the presence of dizziness and associated neck pain. There are no definitive clinical or laboratory tests for CGD and therefore CGD is a diagnosis of exclusion. It can be difficult for healthcare professionals to differentiate CGD from other vestibular, medical and vascular disorders that cause dizziness, requiring a high level of skill and a thorough understanding of the proper tests and measures to accurately rule in or rule out competing diagnoses. Consequently, the purpose of this paper is to provide a systematic diagnostic approach to enable healthcare providers to accurately diagnose CGD. This narrative will outline a stepwise process for evaluating patients who may have CGD and provide steps to exclude diagnoses that can present with symptoms similar to those seen in CGD, including central and peripheral vestibular disorders, vestibular migraine, labyrinthine concussion, cervical arterial dysfunction, and whiplash associated disorder.

## Background

CGD was first described as ‘cervical vertigo’ by Ryan and Cope in 1955, and has at times been considered a controversial diagnosis [1]. The condition has also been named *proprioceptive vertigo*, *cervicogenic vertigo*, and *cervical dizziness*; however, since true vertigo is rarely a symptom seen in people with CGD, it is now generally termed cervicogenic dizziness [2].

Cervicogenic dizziness is characterized by the presence of imbalance, unsteadiness, disorientation, neck pain, limited cervical range of motion (ROM), and may be accompanied by a headache [2, 3]. The cervical spine may be considered the cause of the dizziness when all other potential causes of dizziness are excluded. To be considered CGD, dizziness should be closely related to changes in cervical spine position or cervical joint movement [4]. Although the etiology remains unknown, many cases of CGD have been diagnosed post whiplash injury, or have been associated with inflammatory, degenerative, or mechanical dysfunctions of the cervical spine [5, 6].

What causes the symptoms of imbalance, unsteadiness, and disorientation is not fully understood. Some have suggested the presence of faulty cervical proprioceptive inputs as a contributing factor [7]. It has been proposed that a disruption of the normal afferent signals from the upper cervical proprioceptors to the vestibular nucleus results in an inaccurate depiction of head and neck orientation in space [8]. Another possible cause of these abnormal afferent signals is pain [6].

At present, CGD is a diagnosis of exclusion. A diagnosis of exclusion exists in situations where no single test is able to diagnose the condition, and the diagnosis cannot be verified by outcomes, imaging, laboratory values, or unique signs and/or symptoms [9]. Diagnoses of exclusion are challenging for health practitioners because they require high levels of clinical skill and a strong understanding of the sequencing of proper tests and measures needed to rule out or rule in competing diagnoses. There are many causes of dizziness, including numerous medications and a diverse assortment of vestibular, cardiovascular, metabolic, neurological, psychological, and vision problems. Therefore, a thorough, stepwise process for excluding diagnoses with symptom presentation similar to CGD would be a clinically useful tool for the differential diagnosis of CGD.

Reneker and colleagues [10] conducted a Delphi study to assess the perceived utility of different clinical tests for differentiating between cervicogenic and other causes of dizziness after a sports-related concussion. The authors found no consensus among health practitioners regarding the appropriate tests to identify CGD. The lack of consensus regarding the tests for CGD was cross-professional. Considering the enigmatic nature of CGD, a systematic process is a pragmatic tool for differential diagnosis of CGD. The aim of this narrative is to provide a stepwise process toward the diagnosis of CGD, with utilization of a rule out, rule in paradigm. The determination of which assessment tools to utilize and the order in which the examination is performed is at the discretion of the clinician.

## Main text

**Fig. 1:**
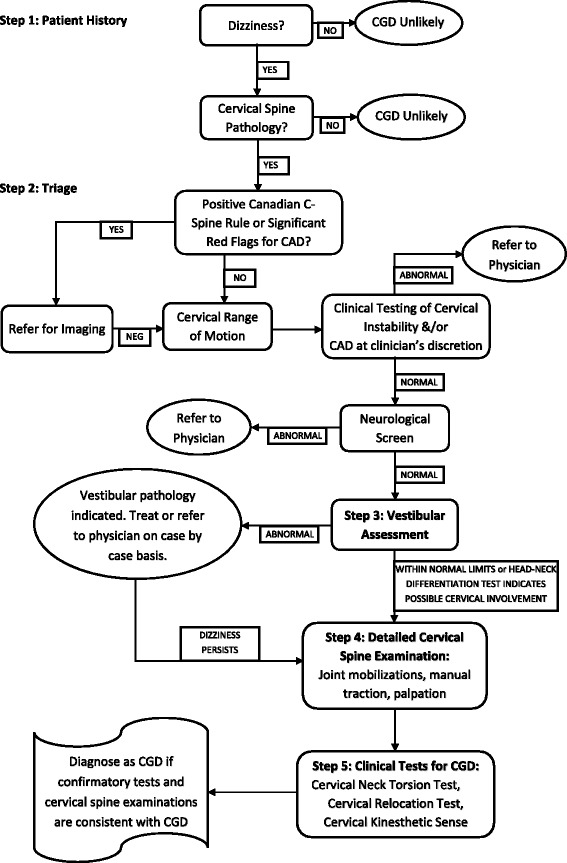
Stepwise algorithm for diagnosing cervicogenic dizziness

Our proposed clinical reasoning stepwise process for diagnosing CGD is depicted in Fig. 1. To rule out competing diagnoses, one needs tests that have low negative likelihood ratios (LR-) and subsequent high sensitivity in order to decrease the post-test probability of the condition when the finding is negative. In contrast, tests that have high positive likelihood ratios (LR+) and subsequent high specificity are used to rule in a condition. The sensitivities, specificities, and likelihood ratios of relevant tests are listed in Table 1. Descriptions and explanations of the tests are listed in Table 2. The background information and details of each step are presented in the following sections.

**Table 1:** Diagnostic accuracy of tests

Test	Diagnosis	Sensitivity (%)	Specificity (%)	PV+ (%)	PV- (%)	LR+	LR-	Reference
Canadian C-Spine Rule	Cervical Spine Trauma	99	45	6.03	100	1.81	0.01	Stiell et al. [36]; Duane et al. [37]
Cervical Arterial Dysfunction (CAD) Test	CAD	0–57	87–100	0–100	26–96	0.22–83.3	0.44–1.4	Hutting et al. [22]; Petersen et al. [38]
Sharp Purser Test	Transverse Ligament Stability	69	96	85	90	15.6	0.33	Uitvlugt & Indenbaum [39]; Hutting et al. [40]
Alar Ligament Test	Alar Ligament Stability	Right: 69 Left: 72	Right: 100 Left: 96	Right: 100 Left: 93	Right: 80 Left: 81	–	–	Kaale et al. [41]
Dix-Hallpike	Posterior Canal BPPV	79.3	75	95.8	33.3	3.17	0.14	Halker et al. [42]
Head Thrust Test	Unilateral Vestibular Hypofunction	71 (88 for complete loss)	82	87	65	4.16	0.3	Schubert et al. [43]
Manual Spinal Examination	Cervical Facet Joint Dysfunction	92	71	–	–	3.17	0.11	Schneider et al. [30]
Palpation for Segmental Tenderness	Cervical Facet Joint Mediated Pain	94	73	–	–	3.48	0.08	Schneider et al. [30]
Cervical Neck Torsion Test	Cervicogenic Dizziness (versus BPPV)	72	92	–	–	9	0.3	L’Heureux-Leabeau et al. [14]
Cervical Relocation Test (with positive test defined by any of the 4 positions with JPE >4.5°)	Cervicogenic Dizziness (versus BPPV)	92	54	–	–	2	0.15	L’Heureux-Leabeau et al. [14]
Cervical Relocation Test (with positive test defined by mean JPE >4.5°)	Cervicogenic Dizziness (versus BPPV)	72	75	–	–	2.9	0.37	L’Heureux-Leabeau et al. [14]
Smooth Pursuit Neck Torsion (SPNT) Test	CGD in people with WAD and dizziness	90 27 56	91 55 88	92 -- --	71 -- --	10 0.6 --	0.11 1.3 --	L’Heureux-Leabeau et al. [14] Tjell & Rosenhall [34] Kongsted et al. [35]

**Table 2:** Test descriptions

Diagnosis	Test	Performance description	Explanation
Traumatic Cervical Spine Injury	Canadian C-Spine Rule [44]	1) Any high-risk factor present: Age ≥ 65 years OR Dangerous mechanism* of injury OR Paresthesias in extremities. If YES to any, radiography should be performed. If NO to all, continue to 2. 2) Any low risk factor that allows safe assessment of range of motion? If NO to all, radiography should be performed; if YES to any, continue to 3. Low risk factors defined as: Simple rear-end motor vehicle collision† OR Sitting position in emergency department OR Ambulatory at any time OR Delayed (not immediate) onset of neck pain OR Absence of midline cervical spine tenderness. 3) Able to actively rotate neck 45° left and right? If unable, radiography should be performed. *Fall from elevation ≥0.9 m (3 ft)/five stairs, axial load to head, motor vehicle collision at high speed (>100 km/h), rollover, ejection, motorized recreational vehicles, bicycle struck, or bicycle collision. †Excludes: pushed into oncoming traffic, hit by bus or large truck, rollover, and hit by high speed vehicle.	Canadian C-Spine Rule is a tool to help clinicians decide if radiography should be utilized in patients following traumatic injury. It is only applicable to patients who are alert (Glasgow Coma Scale score ≥ 15) and in stable condition following trauma where cervical spine injury is a concern. Canadian C-Spine Rule is not applicable in non-trauma cases, for patients with age < 16 years, during pregnancy, or for patients with unstable vital signs, acute paralysis, known vertebral disease, or previous history of cervical spine surgery.
Upper Cervical Instability	Alar Ligament Test [45]	1) Patient assumes sitting or supine position with head slightly flexed to engage the Alar ligament. The clinician assesses the patient’s resting symptoms. 2) The clinician firmly stabilizes the spinous process of C2 using a pincer grasp. 3) Either lateral flexion or rotation is passively performed by the clinician (both are performed independently of each other, in either order). While performing these passive movements, the examiner attempts to feel movement of C2. 4) A positive test is defined by lack of palpable movement of the C2 spinous process during lateral flexion or rotation.	The purpose of this test is to examine the integrity of the alar ligaments following traumatic injury involving the cervical spine. If the alar ligaments are intact, lateral flexion or rotation of the head should result in palpable contralateral movement of the C2 spinous process. Caution is of utmost importance when administering this test.
Upper Cervical Instability	Sharp Purser Test [39, 45]	1) The patient assumes a sitting position with their head slightly flexed. The clinician assesses the patient’s resting symptoms. 2) The clinician stands to one side of the patient and stabilizes the C2 spinous process using a pincer grasp. 3) The clinician uses the opposite hand to gently apply an anterior to posterior translation force on the patient’s forehead. 4) A positive test is defined by symptom reproduction during forward flexion, decrease in symptoms during posterior translation, or excessive displacement (>4 mm) during posterior translation.	This test assesses the integrity of the transverse ligament that maintains the position of the odontoid process relative to C1. If the transverse ligament is torn, C1 will translate forward on C2 during flexion, indicating atlantoaxial subluxation. Atlantoaxial subluxation is the most common cervical spine complication of rheumatoid arthritis. Spinal cord compression secondary to atlantoaxial subluxation can result in severe neurological damage, including quadriplegia and fatality. Extreme caution should be used when administering this test.
Cervical Facet Joint Dysfunction	Manual Spinal Examination [30]	Patient positioned in prone with neutral cervical spine. Clinician applies posterior to anterior directed force to the articular pillars of the cervical spine bilaterally, one joint at a time. In a study by Schneider, et al., a positive test was defined as patient report of ≥3/10 increase in concordant local or referred pain intensity when clinician rated resistance to motion as ‘moderate’ to ‘marked’.	Cervical facet joint capsules contain several sensory receptors including free nerve endings, mechanoreceptors, A-delta and C-fibers, making the joints nociceptive and sensitive to pressure and mechanical changes.
Cervical Facet Joint Mediated Pain	Palpation for Segmental Tenderness [30]	Patient positioned in prone. Clinician palpates deep segmental muscles overlying cervical spine facet joints bilaterally. Schneider, et al. defined a positive test as patient report of ≥3/10 increase in concordant local or referred pain intensity rating.	Segmental muscles overlying painful facet joints often react with tenderness and spasm. Cervical facet joints and the muscles overlying them are innervated by the medial branch of the dorsal rami.
Cervical Arterial Dysfunction	CAD Testing [22, 46]	CAD testing should include the following sequential tests: 1) While seated, the patient performs end range active cervical rotation in both directions. 2) While seated, the patient performs active end range combined cervical extension and rotation in both directions 3) With patient supine, the clinician brings the patient into passive end range cervical rotation in both directions. 4) With patient supine, the clinician brings the patient into passive end range combined cervical extension and rotation in both directions. 5) Any position that the patient reports as provocative. All positions should be held for a minimum of 10 s, unless symptoms are provoked sooner. After each sustained position, the patient should return to neutral cervical spine position for at least 10 s to allow for any latent response to emerge. Throughout CAD testing, the clinician should observe the patient’s eyes for nystagmus, and the patient should report any provocation of symptoms. Positive signs and symptoms include dizziness, nystagmus, diplopia, loss of consciousness, diaphoresis, dysphagia, dysarthria, nausea, numbness around the lips, or other neurological symptoms.	CAD testing involves neck rotation and extension with a stationary body, causing decreased blood flow in the vertebrobasilar arteries with rotation alone and internal carotid arteries with combined extension and rotation. CAD testing requires cervical extension and rotation passive range of motion that is within normal limits.
Vestibular Hypofunction	Head Thrust Test [43]	Grasp the patient’s head firmly with both hands and pitch their head forward 30° to align the horizontal semicircular canals. Instruct the patient to look at your nose. Gently move the patient’s head back and forth with intermittent high velocity, randomly timed thrusts.	While performing head thrusts, observe the patient’s eyes to determine whether they are able to maintain ocular fixation on your nose or not. A failure to maintain fixation on the visual target (nose) indicates hypofunction on the side that the thrust was directed toward. A refixation saccade will be visible for patients who are unable to maintain visual fixation. This test is most valid if the thrusts are performed with random timing that does not allow anticipatory compensation.
Peripheral Vestibular Dysfunction	Head Shaking Induced Nystagmus [47]	Grasp the patient’s head firmly with both hands and pitch their head forward 30° to align the horizontal semicircular canals. Instruct the patient to close their eyes. Passively oscillate the patient’s head side to side 20 times at 1–2 Hz. Instruct them to open their eyes just prior to completing the 20 side to side movements. Observe for post-headshake nystagmus.	The direction of the fast phase of nystagmus denotes the side of higher vestibular functioning. Therefore, the side of vestibular hypofunction is on the side contralateral to the direction of the fast phase.
Benign Paroxysmal Positional Vertigo	Dix-Hallpike Test [42]	The patient is initially sitting upright with legs extended. The clinician passively rotates the patient’s head 45° toward the side being tested. The clinician helps the patient to rapidly lie down on the table while keeping the head slightly extended. The clinician observes the patient’s eyes for nystagmus for at least 60 s; there can be a latency period of up to 15 s before the onset of nystagmus.	The direction of the nystagmus beats will be on the same side as the involved canal. With right sided BPPV, for example, the fast phase of nystagmus will beat to the right with a slow saccade back to the left. Patients with posterior canal BPPV will have a positive Dix-Hallpike test and concomitant vertigo.
Horizontal Canalilithiasis or Cupulolithiasis	Head Roll Test [48]	The patient is initially positioned in supine with their neck flexed 20°. The clinician quickly rotates the patient’s head 90° to either side and observes for nystagmus for at least 60 s. The clinician slowly returns the patient’s head to midline, maintaining neck flexion, then repeats the procedure on other side.	Like the Dix- Hallpike, the nystagmus will beat towards the affected ear with a slow saccade moving in the opposite direction following the fast beat of nystagmus. When the head is rolled toward the affected ear, the nystagmus beats will be in a geotropic (toward the ground) manner. If the head is maintained in this position, a burst of fast beating nystagmus will occur in an ageotropic (away from the ground) fashion. When the head is rolled away from the affected ear, the nystagmus beats will be less intense and in the geotropic fashion.
Vestibular Dysfunction, Cervicogenic Dizziness	Head-Neck Differentiation Test [26–28]	The patient begins seated on a swivel chair. The clinician rotates the chair both while the stabilizing patient’s head and the patient reports any provocation of dizziness. The clinician then rotates the chair without stabilizing the patient’s head and the patient again reports any provocation of dizziness.	Provocation of dizziness with trunk rotation under a stabilized head implicates the cervical spine, whereas dizziness with head and trunk rotation together (*en bloc* rotation) indicates a vestibular component to the patient’s symptoms. If symptoms are provoked in both scenarios, it is likely that CGD and vestibular dysfunction are comorbid. Dizziness of vertebral origin should be ruled out prior to administration of the Head-Neck Differentiation Test.
Cervicogenic Dizziness	Cervical Neck Torsion Test [14]	The patient begins seated on a swivel chair and turns their trunk 90° to the either the right or left, holding for 30 s, then returns their trunk to center. The patient then repeats the same process in the opposite direction. Each position, including the center positions, is maintained for 30 s. Throughout the test, the head is stabilized by the clinician and therefore motionless. The clinician also must continuously observe for nystagmus.	Considered positive if nystagmus (excluding spontaneous nystagmus) of more than 2° per second is observed in any of the four positions (left trunk rotation, neutral rotation, right trunk rotation, neutral rotation).
Cervicogenic Dizziness, Whiplash Associated Disorder	Smooth Pursuit Neck Torsion Test (SPNT) [34, 49]	Surface electrodes are placed on the subject’s skin just lateral to the eyes bilaterally to record the corneo-retinal potential. The subject begins seated with their cervical spine in neutral position. The subject watches a visual target (LED or laser light) that moves through a 40° arc at a frequency of 0.2 Hz with a peak velocity of 20° per second. The subject is instructed to keep their head still and try not to blink while following the light closely with their eyes. The examiner gently holds the subject’s head in place. This process is then repeated with the subject’s body rotated 45° to one side with the head remaining in the same position to create cervical torsion. If 45° of trunk rotation causes discomfort, the angle can be decreased to symptom free range (minimum of 30°). The examiner gently holds the subject’s head and trunk in the position. The test is performed to the left and right sides. The mean gain (i.e. the ratio between eye velocity and target velocity) is calculated in all three positions.	There is a lack of consensus in the description of proper performance methodology of the SPNT. The methods described are based on the initial study of the SPNT performed by Tjell and Rosenhall in 1998. The SPNT is a test of smooth pursuit eye movement with cervical neck torsion. The SPNT is the average value of the smooth pursuit in both the right and left trunk-rotation positions. The difference between the smooth pursuit and the smooth pursuit with neck torsion values is called the smooth pursuit neck torsion difference. The larger the difference between smooth pursuit with neck torsion and smooth pursuit in neutral, the more likely the patient is suffering from a whiplash associated disorder. The utility of the SPNT as a diagnostic tool for differentiating CGD from WAD has been studied in controlled laboratory trials, with mixed results, but has not yet been studied in a clinical setting.
Cervicocephalic Proprioception and Neck Reposition Sense	Cervical Relocation Test [32]	The patient begins seated, facing a wall 90 cm away, and wearing a head-mounted laser pointer that is centered on a target on the wall. The patient keeps their eyes closed while moving their neck in a specified direction, then back to what they believe to be centered starting position. The patient verbally indicates when they believe they are back to center. The patient repeats this process for right rotation, left rotation, flexion, and extension (in no particular order).	The mean distance from the actual center to the subjective center is used to calculate the joint position error (JPE) for each movement. An error of 4.5° is the cutoff point suggesting a failure of head and neck relocalization precision.

### Stepwise process for diagnosing cervicogenic dizziness

#### Step 1: Patient history

In order to determine whether a patient potentially has CGD, it is essential to clarify the symptoms and nature of onset. For CGD to be considered, the patient should have a history of neck pathology and also experience dizziness that has a close temporal relationship with the onset of cervical spine symptoms. Cervicogenic dizziness should not be considered if the patient does not have neck pain. The neck pain can occur at rest, with movement, or with palpation. Symptoms caused by CGD should be exacerbated by movements that elicit neck pain and should subside with interventions that alleviate neck pain.

It is imperative to obtain a thorough patient history as the first step in the diagnostic process in order to identify red flags, to begin ruling out competing pathologies, and to prioritize pathologies that best fit the description of the onset, signs, and symptoms. Table 3 details the typical clinical presentations of CGD and the pathologies that can present with similar symptoms. Important information to seek for patients with both dizziness and neck pain includes presence of cardiovascular risk factors, history of migraines, symptoms of tinnitus or aural fullness, oscillopsia, and symptoms exacerbated by exertion, positional changes, busy environments, or specific activities.

**Table 3:** Presentation of cervicogenic dizziness and competing diagnoses

Diagnosis	Duration	Signs and symptoms
Acute Vestibular Loss	Single attack, several attacks, or persistent for several weeks.	Sudden vertigo or dizziness possibly accompanied by tinnitus, diplopia, nausea, vomiting [15].
Benign Paroxysmal Positional Vertigo	A few seconds to several minutes.	Vertigo. Occurs with changes in position relative to gravity [50].
Central Vestibular Disorders	Several days to weeks.	Constant vertigo, facial asymmetry, swallowing or speech problems, ptosis, ataxia, sensation changes, upper motor neuron signs, abnormal head thrust test, direction changing nystagmus, pure vertical nystagmus, pure torsional nystagmus, a skew deviation, and other neurological symptoms [23, 51].
Cervical Arterial Dysfunction	Several minutes.	Dizziness that is typically accompanied by diplopia, numbness around the lips, nystagmus, ataxia, bilateral neurological symptoms, dysphagia, dysarthria and headaches. Associated with nausea and vomiting [19].
Cervicogenic Dizziness	Several minutes to hours [3].	Dizziness and disequilibrium due to changes in cervical spine position [3].
Labyrinthine Concussion	Episodically over hours to days [26].	Cervical neck pain is common. Hearing loss, tinnitus, and dizziness [18].
Ménière’s Disease	Minutes to hours, rarely longer than 24 h [12].	Presents with episodic, intense vertigo, accompanied by aural fullness, tinnitusand fluctuating hearing loss. Attacks are typically preceded by aura and followed by a period of exhaustion and generalized dizziness. As Ménière’s disease progresses, the hearing loss and tinnitus intensify and become more persistent, and the acute attacks of vertigo may be replaced by more chronic problems with dizziness and imbalance [12, 13].
Vestibular Migraine	4–72 h.	Vestibular Migraine Diagnostic Criteria (International Headache Society) [17]. A. At least five episodes involving criteria C and D. B. A current or past history of *Migraine without aura* or *Migraine with aura.* C. Vestibular symptoms* of moderate or severe intensity, lasting between 5 min and 72 h. D. At least 50% of episodes are associated with at least one of the following three migrainous features: 1) headache with at least two of the following four characteristics: unilateral location, pulsating quality, moderate or severe intensity, aggravation by routine physical activity; 2) photophobia and phonophobia; 3) visual aura. E. Not better accounted for by another ICHD-3 diagnosis or by another vestibular disorder. *Barany Society’s Classification of Vestibular Symptoms: a. spontaneous vertigo: i. internal vertigo (a false sensation of self-motion) ii. external vertigo (a false sensation of visual surroundings spinning or flowing) b. positional vertigo, triggered by a complex or large moving stimulus c. visually induced vertigo, triggered by a complex or large moving visual stimulus d. head motion-induced vertigo, occurring during head motion e. head motion-induced dizziness with nausea
Whiplash Associated Disorder	Variable. Days to weeks and in some cases months.	Cervical neck pain and hypersensitivity, decreased cervical range of motion, dizziness, tinnitus, and headache. Associated with psychological factors, such as low pain tolerance and fear avoidance [21].

The nature of the patient’s symptoms can aide in determining the cause of dizziness. It is important to clarify the quality of the reported “vertigo” or “dizziness,” as there is significant inconsistency in the use of these terms. Dorland’s Illustrated Medical Dictionary [11] defines vertigo as an illusion of movement; a sensation as if the external world were revolving around the individual (objective vertigo), or as if the individual were revolving in space (subjective vertigo). Vertigo is not a symptom arising from the cervical spine, but rather is caused by peripheral vestibular disorders or lesions within the vestibular pathways of the central nervous system.

The duration of symptoms is another important aspect of the subjective history that helps differentiate CGD from other pathologies. The duration of symptoms for CGD can range from days to months to years. Each episode of dizziness typically lasts minutes to hours [3]. The typical duration of symptoms for each pathology discussed can be found in Table 3.

There are numerous vestibular causes of dizziness with characteristic presentations that can help distinguish each from CGD. Ménière’s disease is a chronic vestibular disorder characterized by episodic bouts of aural fullness, vertigo, and hearing loss [12, 13]. Cervicogenic dizziness however, typically does not include aural fullness, tinnitus, or hearing loss. The presence of these symptoms does not definitively rule in Ménière’s disease, but does decrease the likelihood that CGD is implicated.

Benign paroxysmal positional vertigo (BPPV) is the most common vestibular pathology. BPPV occurs when calcium carbonate crystals (otoconia) become dislodged from the utricle and migrate into one of the three semicircular canals located within the inner ear. BPPV presents as vertigo associated with changes in head position relative to gravity. The vertigo associated with BPPV is accompanied by nystagmus. The most common pattern of nystagmus seen in BPPV is a mixed up-beating and torsional nystagmus, but the direction of the nystagmus will vary depending on which semicircular canal is affected. If a patient has symptoms of vertigo accompanied by nystagmus in response to changing head position in space, then BPPV is more likely than CGD. In a study comparing BPPV to CGD, sensations of “drunkenness” and “fainting” were found in both groups, however these symptoms were reported significantly more frequently by the CGD group than the BPPV group, whereas rotatory sensation and symptom duration lasting only a few seconds were significantly more common in the BPPV group [14]. Cervical neck movement, fatigue, anxiety, and stress were also found to be more common precipitating factors for exacerbation of symptoms in the CGD group as compared to the BPPV group.

A patient with an acute, unilateral, peripheral vestibular loss due to labyrinthitis or vestibular neuronitis will generally present with marked vertigo and imbalance, with associated nausea and possibly vomiting. In addition, the patient will acutely present with spontaneous horizontal, direction fixed nystagmus in room light. As the acute signs and symptoms dissipate, the patient may be left with a variety of symptoms, including dizziness, motion sensitivity, imbalance, difficulty with concentration, tinnitus, and hearing loss [15]. The subjective report is useful in differentiating vestibular loss from CGD because patients with CGD typically do not have tinnitus, hearing loss or vertigo.

Vestibular migraines have been described as the most common cause of recurrent vertigo [16]. Refer to Table 3 for the specific criteria developed by The International Headache Society to diagnose a vestibular migraine. Key symptoms frequently present in vestibular migraine, but not CGD, are aura, true vertigo, throbbing headaches, sensitivity to auditory or visual sensory stimulation, and oculomotor changes [17]. Patients with vestibular migraine do not typically experience dizziness related to mechanical neck pain or dysfunction.

Labyrinthine concussion is a vestibular disorder that can mimic symptoms of CGD due to the presence of dizziness and cervical neck pain [3]. Distinguishing between CGD and labyrinthine concussion is complicated by the fact that both labyrinthine concussion and CGD can be attributed to trauma. Labyrinthine concussion often includes signs and symptoms similar to those of a peripheral vestibular loss (as described above), including hearing loss, tinnitus and dizziness [18].

Cervical arterial dysfunction (CAD) and whiplash associated disorder (WAD) are non-vestibular pathologies that can mimic CGD. Cervical arterial dysfunction is a term sometimes incorrectly used interchangeably with vertebrobasilar insufficiency (VBI). However, VBI refers only to decreased blood flow in the vertebrobasilar arteries, whereas CAD refers to restricted blood flow in any of the cervical arteries, including the internal carotid arteries [19]. There are many potential underlying causes of CAD, including but not limited to atherosclerosis, thrombosis, pre-existing anatomical anomalies, cervical arterial dissection, vasospasm, and external compromise. The clinician should determine if the patient has any cardiovascular risk factors that may increase their likelihood of CAD, such as hypertension, hypercholesterolemia, blood clotting disorders, diabetes mellitus, smoking, vessel trauma, or history of cardiac or vascular disease [19]. CAD presents as dizziness lasting several minutes that is related to movements of the head on the trunk [19]. If the patient reports dizziness without other CAD symptoms, it is very unlikely that CAD is the cause, as CAD related dizziness presents with only one symptom in less than 1% of cases [20]. Other symptoms of CAD include severe headache, diplopia, nystagmus, numbness around lips or mouth, dysphagia, dysarthria, and upper motor neuron signs [19].

Whiplash associated disorder develops after a trauma involving rapid acceleration and/or deceleration, most commonly a motor vehicle accident. Patients with WAD typically have low pain tolerance and score high on fear avoidance measures such as the Fear Avoidance Belief Questionnaire (FABQ). Common symptoms of WAD are cervical neck pain and hypersensitivity, decreased cervical ROM, dizziness, tinnitus, and headache [21]. In some cases, the dizziness that accompanies WAD may be CGD. WAD can also be associated with a concussion that occurs during a traumatic injury. Therefore, WAD, concussion, and CGD are not mutually exclusive diagnoses. When a person experiences dizziness as a symptom of concussion and/or WAD, the dizziness can be of cervical origin.

#### Step 2: Triage

If the subjective report includes blunt trauma, triage of the cervical spine should first utilize the Canadian C-Spine (cervical spine) Rule to determine if radiography is indicated. The criteria for the Canadian C-Spine Rule are detailed in Table 2. The Canadian C-Spine Rule has high sensitivity, and therefore it is very unlikely to incorrectly determine that a patient with a severe cervical spine injury does not require radiography. If the patient reports significant red flag symptoms consistent with CAD, they should be referred for diagnostic imaging to rule out the cervical arteries as a potential cause of dizziness. Clinical tests for cervical instability and CAD are provocative in nature and therefore should be used sparingly and with utmost caution. Positive results on cervical instability testing or CAD testing indicate a need for immediate medical attention and imaging [22].

If no imaging is warranted based on the Canadian C-Spine Rule or significant red flag symptoms of CAD, the clinician should proceed with assessment of cervical range of motion. Cervical ROM is appropriate at this juncture in the triage stage because several tests that the clinician may utilize later in the examination, including vestibular tests discussed in Step 3, have minimum cervical ROM requirements. Furthermore, identifying limitations or symptom provocation with active or passive cervical ROM is an efficient way for the clinician to gain useful information prior to embarking on more complex vestibular and cervical spine testing. For example, the cervical neck torsion test only discriminates using rotation, so if a patient’s dizziness is provoked by extension ROM rather than rotation, the clinician may prioritize other clinical tests over the cervical neck torsion test.

Given that patients being evaluated for CGD present with an unknown cause of dizziness and were often involved in a traumatic event, a neurological screen will likely be indicated. A neurological screen should include an assessment of radicular symptoms, myotomes, dermatomes, deep-tendon reflexes, upper motor neuron signs, and cranial nerve function. Abnormal neurological findings may warrant referral to either a neurologist or emergency care for further evaluation, depending on the severity. Central vestibular disorders can present with a variety of symptoms, ranging from constant vertigo to generalized symptoms of dizziness, and will typically present with red flag signs and symptoms that warrant referral to a physician [23].

If cervical instability, CAD, and neurological dysfunction are ruled out, the clinician should proceed with clinical tests to rule out vestibular pathologies.

#### Step 3: Vestibular assessment

If a patient has a history consistent with CGD and has been properly screened in the triage stage, the vestibular system should be assessed next. All patients should have a rudimentary cervical spine examination prior to vestibular testing, including subjective report of cervical spine pain established in Step 1, as well as assessment of cervical spine ROM and radicular symptoms in Step 2. The vestibular exam can be modified to limit the head movements to the available cervical ROM. A thorough evaluation of the cervical spine is best performed in Step 4 because first ruling out vestibular dysfunction increases the probability that the cervical spine is the cause of dizziness. With that being said, there is likely to be some overlap between Step 3 and Step 4; the nature of the presenting history, symptoms and signs will dictate the order of evaluation and treatment. In the case of obvious vestibular causes of dizziness (e.g. BPPV, vestibular hypofunction) without acute cervical spine involvement, treatment of the vestibular pathology would be initiated prior to moving on to Step 4. If there is markedly restricted cervical spine ROM that precludes treatment of the vestibular pathology, then cervical spine assessment and treatment would have to precede (or occur concurrently with) the treatment of the vestibular pathology.

Within the vestibular functioning step, oculomotor evaluation should include evaluation of nystagmus, skew, smooth pursuit, saccades, Dix-Hallpike test, static and dynamic visual acuity, and the vestibulo-ocular reflex (VOR) including VOR cancellation and the head thrust test. The observation of nystagmus is clinically useful to determine if the vestibular system is involved, and the presence of nystagmus during testing can help to rule out CGD.

A horizontal, direction fixed nystagmus is consistent with unilateral peripheral vestibular hypofunction. Patients with unilateral vestibular hypofunction typically have oculomotor signs such as a positive head thrust test or head shaking induced nystagmus, and may have abnormal dynamic visual acuity—these findings would not typically be seen in an individual with CGD. The absence of spontaneous or gaze-evoked nystagmus in room light does not rule out a peripheral vestibular deficit because patients with peripheral vestibular hypofunction can utilize visual fixation to suppress nystagmus. Therefore, utilization of Frenzel lenses allows for more reliable detection of unilateral peripheral vestibular hypofunction as the Frenzel lenses will remove visual fixation. Individuals who have compensated for a unilateral loss will often have no nystagmus in room light, whereas individuals with bilateral vestibular loss generally have no nystagmus in either room light or with visual fixation removed. Nystagmus originating from a central pathology demonstrates a different pattern; the nystagmus will be present in room light and will either persist or diminish when visual fixation is removed. Direction changing nystagmus, pure vertical nystagmus, or torsional nystagmus is consistent with a central vestibular deficit.

Other oculomotor abnormalities, such as saccadic smooth pursuit or saccadic abnormalities, may be seen in patients with central vestibular and central oculomotor deficits. While there have been some reports of abnormal eye movements in cases of WAD, the results from different studies are highly variable [24, 25]. There is not a single, definitive oculomotor test that is capable of identifying CGD.

Cervicogenic dizziness and dizziness from vestibular disorders can be differentiated using the head-neck differentiation test, which is a variation of the cervical neck torsion test [26–28]. The test is performed with the patient sitting on a swivel chair. Provocation of dizziness with trunk rotation under a head stabilized in space implicates the cervical spine, whereas dizziness with head and trunk rotation together (*en bloc* rotation) indicates a vestibular component to the patient’s symptoms. This test can be performed for both horizontal and pitch plane motions of the head and cervical spine. If symptoms are provoked in both scenarios, it is likely that CGD and vestibular dysfunction are comorbid, and then both the vestibular and cervicogenic components can be addressed.

While static and dynamic balance tests are not diagnostic for vestibular dysfunction, these tests are often abnormal in individuals with vestibular deficits [28, 29]. Studies have also shown that cervical pain can cause decreased standing balance and postural control [8]. Patients with either vestibular dysfunction or CGD may have increased symptoms during a dynamic balance assessment. While not diagnostic for either condition, assessment of static and dynamic balance is important from the perspective of a functional assessment.

Positive results on vestibular tests do not rule out cervicogenic dizziness, as a patient can have two causes of dizziness simultaneously. If a patient is found to have vestibular dysfunction, the clinician may initiate treatment of the dysfunction if it is within their capabilities, as well as refer to an otolaryngolist or neurologist depending on the patient’s presenting signs and symptoms for further medical assessment. If treatment of the vestibular impairment does not lead to complete resolution of the patient’s symptoms of dizziness, or if the head-neck differentiation test indicates cervical and vestibular involvement, the clinician should consider the possibility that the patient has both dizziness of vestibular origin and CGD, and proceed with Step 4.

#### Step 4: Detailed cervical spine evaluation

Although cervical range of motion testing and cervical instability testing are most appropriately performed as part of triage, thorough evaluation of the cervical spine should ideally be performed after vestibular testing in order to rule out vestibular dysfunction and thereby narrow the list of potential causes of dizziness. Cervical spine evaluation includes manual spinal examination (MSE) for facet joint dysfunction, palpation for segmental tenderness (PST), assessment of postural alignment, and traction.

There is no individual test that can reliably diagnose the cervical facet joint as a source of pain. However, in one study, MSE and PST both exhibited high sensitivity (92% and 94%, respectively), demonstrating potential utility as screens for cervical facet joint mediated pain [30]. Manual spinal examination should include unilateral posterior to anterior mobilization of cervical facet joints with assessment of pain provocation and resistance to motion. To perform PST, the clinician palpates the muscles over the cervical facet joints and assesses for increased concordant pain. Individuals with CGD commonly present with tight posterior neck muscles and tenderness of both posterior neck muscles and cervical facet joints. In a study by L’Heureux-Leabeau et al. [14], the CGD group was significantly more likely than the BPPV group to experience pain during physical examination of the upper cervical spine and paravertebral muscles.

Postural alignment and control should be assessed because postural impairments are commonly seen in cases of CGD, especially in cases with neck pain from whiplash injury [8]. A reduction of dizziness symptoms in response to cervical traction implicates involvement of the cervical spine and is more consistent with CGD than with vestibular dysfunction [31]. It is best to perform traction with the patient sitting in order to minimize the effect of gravity on the vestibular system.

#### Step 5: Clinical tests for cervicogenic dizziness

While CGD is a diagnosis of exclusion and cannot be definitively ruled in with any single test, there are tests that have been demonstrated to be clinically useful. Ruling out competing diagnoses in previous steps will increase the pre-test probability of CGD, thereby increasing the post-test probability when utilizing these clinical tests.

The test with the strongest diagnostic utility to rule in the diagnosis of cervicogenic dizziness is the cervical neck torsion test (LR+ of 9), which measures nystagmus in response to cervical neck rotation [14]. The cervical relocation test, a measure of joint position error, has good diagnostic value for ruling out the diagnosis of cervicogenic dizziness (LR- of 0.15). The diagnostic value of both the cervical neck torsion test and cervical relocation test are limited by the fact that the likelihood ratios are based on a study comparing only CGD and BPPV. L’Heureux-Leabeau et al. [14] found that the cervical neck torsion test and cervical relocation test are most useful for differential diagnosis of BPPV versus CGD when the results of the two tests were combined. Table 2 describes how to perform the cervical neck torsion test and the cervical relocation test.

Revel and colleagues [32] demonstrated that individuals with chronic neck pain have impaired head relocation after active head rotation. Multiple studies of cervical kinesthetic sense have shown that cervical repositioning errors are greater in cases of WAD that include complaints of dizziness, as opposed to WAD cases without dizziness, especially for tests of cervical rotation [33]. While it is unclear whether these results are due to dizziness or pain, impaired cervical kinesthetic sense is important to consider as one aspect of CGD.

The smooth pursuit neck torsion test (SPNT) is a laboratory test that has been proposed for differentiating CGD from WAD. The SPNT test is a comparison of the gain (the ratio of the eye velocity to the target velocity) of the eye response in neutral versus rotated head positions. One laboratory study found the gain difference to be significantly greater in WAD cases that include dizziness, as compared to WAD cases without dizziness [34]. However, other laboratory trials using the SPNT test have concluded that it is not useful for differentiating CGD from WAD [35]. The reliability, validity and diagnostic accuracy of a clinical version of the SPNT for differential diagnosis of CGD has not been determined. At this point, there is no clinical advantage to using the SPNT test alongside cervical neck torsion testing as both the sensitivity and specificity are higher in cervical neck torsion testing [14]. Therefore, the SPNT test is currently not a clinically useful test for diagnosing CGD.

### Study limitations

It is worth noting that the diagnostic utility of many special tests used for the diagnosis of CGD have been studied by comparing two specific populations. For example, differentiating only between BPPV and CGD or between CGD and WAD. Therefore, this paper is limited by the goal of portraying a comprehensive, generalized clinical thought process by combining the insights of a wide variety of studies, each offering conclusions about specific questions. Each test and technique discussed has its own limitations. For example, the most well-known test for CGD is the head-neck differentiation test, which has not yet been studied for diagnostic utility. The clinical tests are each limited by their scope. For example, the cervical neck torsion test only examines dizziness provocation using rotation, so these tests may not reproduce dizziness in patients with CGD that is provoked by movement into other planes. The tests with the most clinically useful likelihood ratios, the cervical neck torsion test and cervical relocation test, were established in a study comparing only CGD and BPPV, and therefore are not sufficiently validated to be considered independently conclusive measures for ruling CGD in or out in the general population. The value of this framework for the diagnosis of CGD has not yet been validated using a controlled clinical trial. This paper is an amalgamation of the current evidence for best-practice in the diagnosis of CGD combined with the opinions of clinical experts (RC).

## Conclusions

Without robust diagnostic tests to definitively diagnose or exclude CGD, it is currently best categorized as a diagnosis of exclusion. To diagnose CGD, masquerading pathologies must be identified and excluded. However, a thorough subjective history and triage screening can narrow the list of potential pathologies. If a patient does not report both dizziness and cervical involvement, CGD is unlikely. CGD is also less likely if the patient reports tinnitus, hearing loss or migraines. Duration of symptoms can further narrow the list of likely pathologies. After obtaining the subjective report, it is sometimes necessary to screen for neck instability and CAD involvement before moving on to clinical tests. Vestibular testing, such as the head-neck differentiation test and Dix-Hallpike maneuver, can then be used to determine if the vestibular system is causing the dizziness. Once vestibular pathologies have been ruled out, the clinician should examine the cervical spine, followed by the cervical neck torsion test and cervical relocation test to help confirm or exclude the diagnosis of CGD. If all other pathologies have been ruled out and the exam results are generally consistent with CGD, the clinician should make the diagnosis of CGD. It is possible for patients to have both CGD and another cause of dizziness, such as WAD or a vestibular pathology. In this scenario, the clinician can be most confident about diagnosing the patient with cervicogenic dizziness after they have thoroughly addressed the comorbidity with appropriate interventions, but dizziness still persists.

## Abbreviations

BPPV: Benign paroxysmal positional vertigo
CAD: Cervical arterial dysfunction
CGD: Cervicogenic dizziness
CTA: Computerized tomography angiography
ERT: Extension-rotation test
FABQ: Fear-avoidance beliefs questionnaire
JPE: Joint position error
LED: Light emitting diode
LR-: Negative likelihood ratio
LR+: Positive likelihood ratio
MRA: Magnetic resonance angiography
MRI: Magnetic resonance imaging
MSE: Manual spinal examination
PST: Palpation for segmental tenderness
PV-: Negative predictive value
PV+: Positive predictive value
ROM: Range of motion
SPNT: Smooth pursuit neck torsion
VBI: Vertebrobasilar insufficiency
VOR: Vestibulo-ocular reflex
WAD: Whiplash associated disorder
